# The smile arc: review and synthesis

**DOI:** 10.1590/2177-6709.26.3.e21spe3

**Published:** 2021-06-30

**Authors:** Máyra Reis SEIXAS, Carlos Alexandre CÂMARA

**Affiliations:** 1 Private practice (Salvador/BA, Brazil).; 2 Private practice (Natal/RN, Brazil).

**Keywords:** Esthetics, Upper incisal line, Smile arc, Orthodontic planning

## Abstract

**Introduction:**

The smile arc is an esthetic parameter that has been better investigated by Orthodontics after the “new esthetic paradigm”. Its diagnostic evaluation and inclusion in the objectives of orthodontic planning has become fundamental for professionals seeking for more beautiful and youthful natural esthetic outcomes.

**Objectives:**

To review concepts related to the smile arc, analyze the determinants of its appearance, understanding how the possible variations can affect the esthetic perception of smile.

## RESUMO

**Introdução:**

O arco do sorriso é um parâmetro estético que passou a ser mais bem estudado pela Ortodontia a partir do “novo paradigma estético”. A sua avaliação diagnóstica e inclusão nos objetivos dos planejamentos ortodônticos tornou-se fundamental para profissionais que buscam resultados estéticos mais belos e joviais.

**Objetivos:**

Revisar conceitos relacionados com o arco do sorriso, analisar as determinantes da sua aparência, e compreender como suas possíveis variações podem repercutir na percepção estética do sorriso.

## INTRODUCTION

The smile is the most pleasant expression on the face, translating the beauty, youth and personality of people. From this understanding, raises one of the objectives of orthodontic treatment: the ability to restore smiles adapted to the face, age and lifestyle of patients, enhancing their positive esthetic characteristics and increasing the self-esteem and self-confidence when smiling.^1^ ^,^ ^2^ ^,^ ^3^

**Figure 1:**
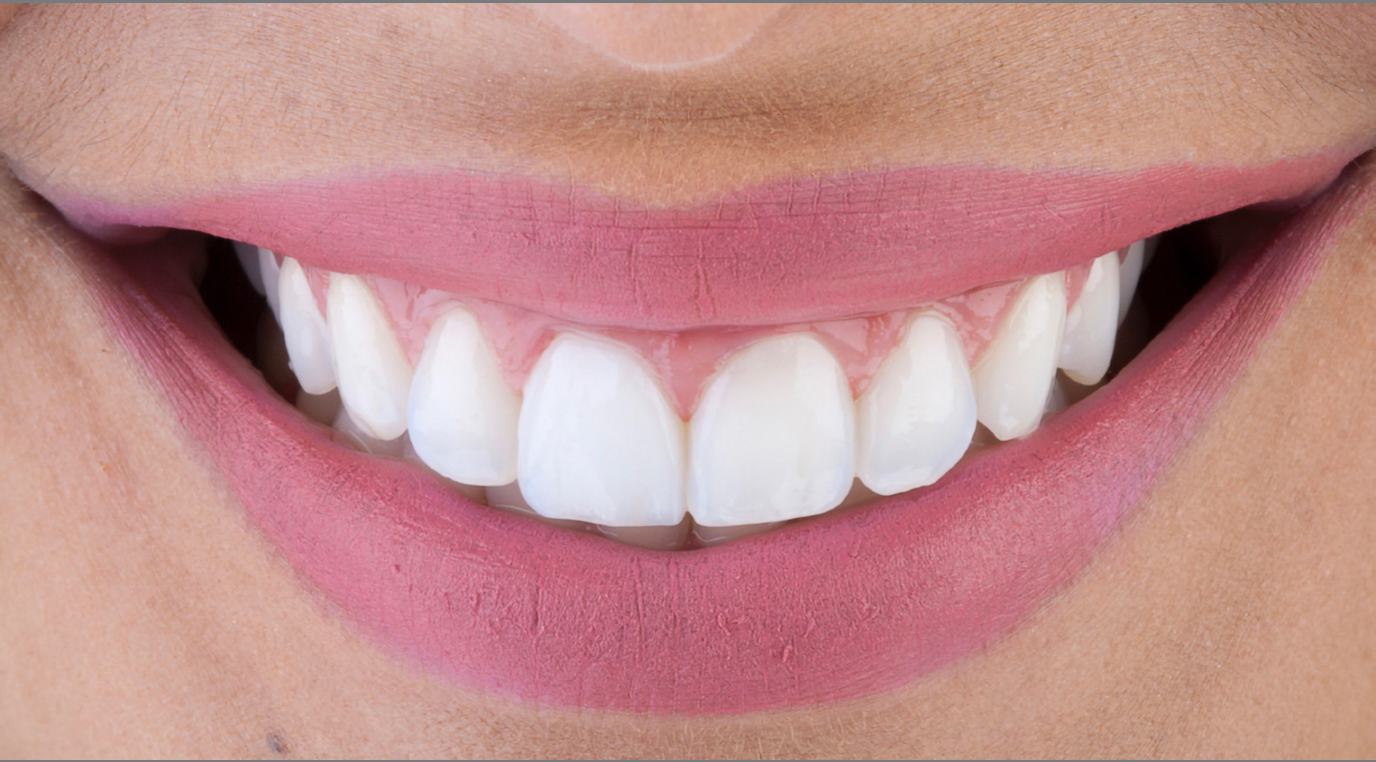
The ideal Smile Arc occurs when the upper incisal line has a similar curvature as that formed by the lower lip when smiling.^4^ ^,^ ^5^

The components that define the maximum esthetic potential have been extensively discussed in the orthodontic literature, many originated from concepts of complete dentures. One of the most important, the “Smile arc” (also known as “Smile curve”), was defined as the relationship between the curvature of the incisal edges of maxillary anterior teeth (upper incisal line) and the curvature formed by the lower lip when smiling (Fig 1).^4^ ^,^ ^5^ This issue has been extensively studied by restorative specialties,^6^ ^-^ ^9^ and has gained the attention of orthodontists, based on the “new esthetic paradigm”,^10^ ^,^ ^11^ which has been guiding treatments since the 1990s.

In August 2001, David Sarver published one of the most read papers of AJODO: *“The importance of incisor positioning in the esthetic smile: The smile arc.”* ^5^ His contribution was mainly based on the findings of some studies that confirmed the lower attractiveness of flatter upper incisal lines, and in studies reporting that this flattening was more common in orthodontically treated patients, compared to individuals with “normal” untreated occlusion.^3^ ^,^ ^12^ ^,^ ^13^ ^,^ ^14^

This publication by Saver^5^ became popular by recognizing the importance and impact of this esthetic parameter on the orthodontic outcomes, in both short and long terms, besides evaluating the resources and mechanical decisions that harm, maintain or optimize its design and appearance. Then, clinical orthodontists began to dedicate more attention to this aspect, since it had been scarcely observed, understood and addressed for a long time.

**Figure 2:**
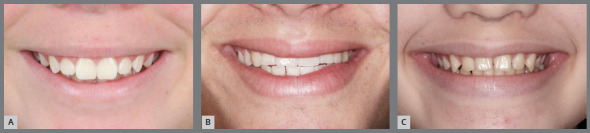
Types of smile arcs: A) pleasant and youthful; B) straight or flat, and C) reverse or inverted.

Several other scientific publications also provided well-known and important contributions on this topic.^15^ ^-^ ^18^ Following are the most known:

» The smile arc was considered as “consonant and pleasant” when it looks younger and presents the curvature of the upper incisal line similar to the curvature formed by the lower lip when smiling (presented in the literature as the esthetically most well accepted) (Fig 2A); “flat”, when presents a flattened upper incisal line in relation to the curvature of the lower lip (Fig 2B); and “inverse”, when presents a more aged appearance, in which the upper incisal line forms an opposite curvature to that formed by the lower lip, during social or voluntary smile (Fig 2C). According to Dong et al.^7^, 60% of patients present smile types “consonant and pleasant”; 34% present arches type “flat” and 5%, “inverted” (Fig 2C).
» It is known that females present a more marked upper incisal line than males, and in both the curvatures formed by the upper incisal line and lower lip when smiling tend to be flatter with the increase in age.
» It has been observed that, during the aging process, the lips assume a lower position, contributing to the reduction of exposure of the maxillary anterior teeth and increased exposure of mandibular anterior teeth.
» Orthodontics acknowledges its limitations when facing problems related to the morphology and behavior of the lips when smiling. The lower lip does not always form a concave curvature to serve as parameter for the convexity of the upper incisal design. ^2,16^ Since the smile arc is formed by the consonance of two curved lines, the isolated establishment of a beautiful curvature of the upper incisal line is insufficient to define its formation and aspect. However, this is the only site of action whose change is within the scope of Orthodontics.

Considering previous publications and using some clinical perception on the issue, this paper aims to organize and summarize the subject, facilitating the diagnosis and understanding of factors that can determine the achievement of different esthetic perceptions in relation to the smile arc.

**Figure 3:**
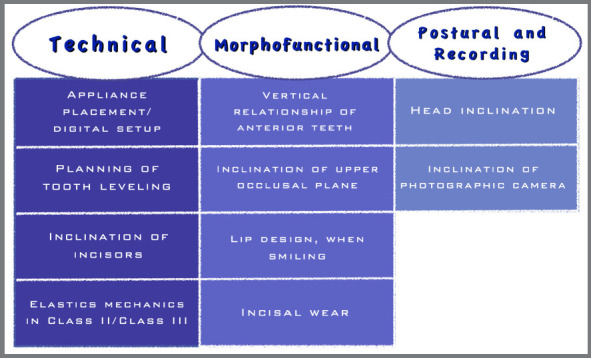
Determinants of the formation and aspect of the Smile Arc can have a technical nature, morphofunctional origin and those related to posture and photographic recording issues.

The determining factors for its formation and aspect can be listed into three main groups, described in Figure 3.

## 1 - TECHNICAL DETERMINANTS

The aspects related to planning and accomplishment of orthodontic or orthodontic-surgical treatment.

**Figure 4:**
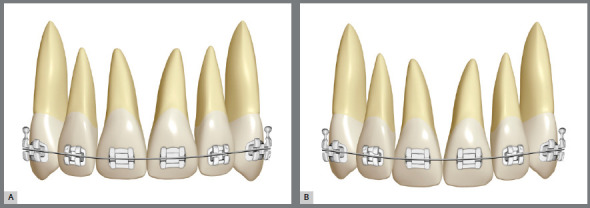
A) Placement of upper appliance with attention focused on achieving the canine guidance: the heights of brackets tend to flatten the smile arc. B) Appliance placement based on the contemporary esthetic paradigm: decreased dominance of cusp tips of maxillary canines and formation of a smooth convex upper incisal line (shaped as a “deep dish”), which allows the formation of an esthetically pleasant smile arc .

**Figure 5:**
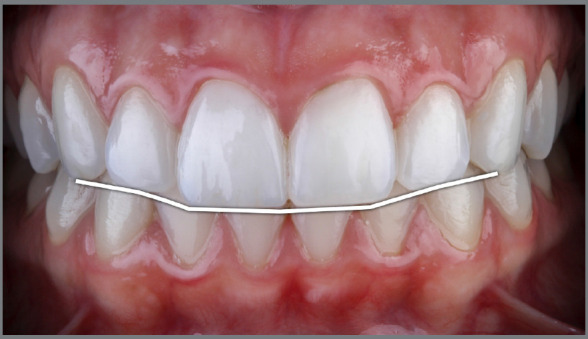
Upper incisal line shaped as a “deep dish”.

**Figure 6:**
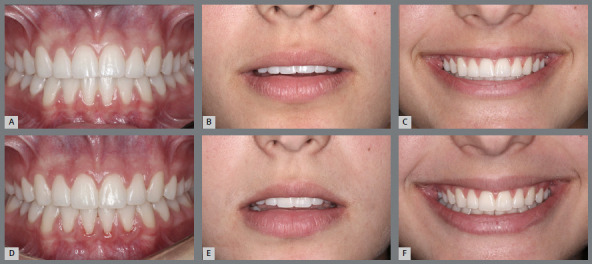
A-C) Intraoral and extraoral photographs of the upper incisal line flattened by orthodontic treatment, forming a straight and not so youthful smile arc for a 15-year-old girl. B) Improvement of the upper incisal design, after a new appliance placement, promoting greater curvature of the upper incisal line and forming a more pleasant, feminine and youthful smile arc.

**Figure 7:**
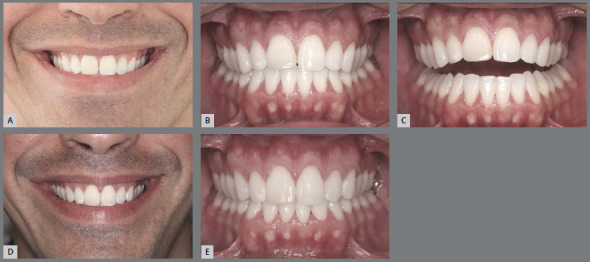
Intraoral and extraoral photographs of the inverted upper incisal line after overbite correction by a sharp curve of Spee in the upper arch (**A**-C). **D,**E) Retreatment of the case, with good control of overbite, reestablishment of the curvature of the upper incisal line and formation of a more pleasant and youthful smile arc.

**Figure 8:**
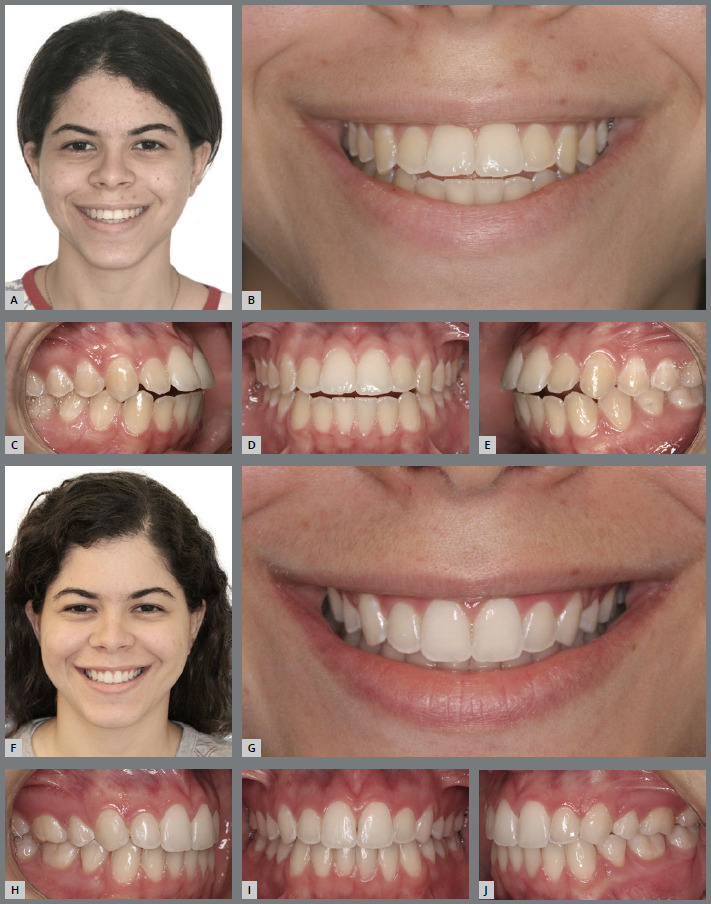
A-E) Patient treated by projection of incisors, presenting dentoalveolar biprotrusion, anterior open bite, flat upper incisal line, and change of light reflection on the buccal surfaces of maxillary incisors. F-J) Retreatment performed by tooth extractions and retraction of anterior teeth: improved light reflection from the smile and achievement of an upper incisal line shaped as a “deep dish”. The correct appliance placement and tooth movement performed enabled the formation of a more esthetically acceptable smile arc.

**Figure 9:**
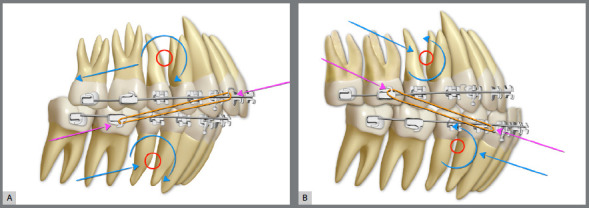
Elastic mechanics applied in Class II (A) and Class III (B) directions, and their possible side effects in the inclination of upper and lower occlusal planes.

»*Appliance placement/Digital setup:* Figuring as the initial step of active treatment, this should be performed aiming at achieving an individualized incisal design, according to the patient’s gender and age.^15^ ^,^ ^19^ ^-^ ^22^ In the recent past, with planning focused almost exclusively on occlusion, the concern with the role of canines in laterality movements defined a more occlusal positioning of their cusp tips, when compared to the incisal edges of upper central incisors (Fig 4A). Currently, the appliances position the edges of maxillary central incisors leveled with the canine cusp tips (Fig 4B), since the achievement of correct disocclusion in laterality can be compensated by the more occlusal positioning of mandibular canines. The edges of lateral incisors should be nearly 1mm below the maxillary central incisors, creating an upper anterior incisal design shaped as a “deep dish” ^2^ ^,^ ^16^ (Fig 5). Concerning factors related to gender and age, female smiles accept an incisal design exhibiting greater height discrepancy between maxillary central and lateral incisors (from 1.0 to 1.5mm), while older patients require less marked incisal designs, with smaller height discrepancies between the upper incisal edges. Figure 6A shows a situation in which the upper incisal line was flattened due to an inadequate appliance placement, and Figure 6B shows its correction after retreatment using a more favorable placement. Also, the perception of canine tips in frontal view is influenced by the distortion of perspective, the known parallax effect (what is more distant seems smaller) and by the occlusal plane inclination, as shown later.^2^ In digital planning, it is possible to use tools to guide the upper incisal design, such as the “Smile Curves” template.^15^
»*Planning of arch leveling:* the correction of vertical problems present in the anterior region of dental arches, as increased overbite and open bite, requires planning based on the ideal exposure of maxillary incisors, in lip rest position.^1^ ^,^ ^5^ ^,^ ^6^ ^,^ ^10^ ^,^ ^12^ ^,^ ^13^ ^,^ ^20^ Currently, an excellent tool for planning of orthodontic leveling is the “Functional Aesthetic Occlusal Plane” published by Câmara and Martins^20^ in 2016.The “FAOP” allows to identify the needs and limits of vertical movement of anterior teeth, favoring the maintenance or achieving the ideal exposure of maxillary incisors in lip rest position. In the past decades, flat and inverted smile arcs, caused by the correction and control of overbite by anterior maxillary teeth intrusion performed with “steps” or marked curves of Spee in the dental arches, were responsible for very undesirable esthetic results regarding the beauty and youthfulness of smile (Fig 7). Therefore, in the search for patients with pleasant and youthful smile arcs, planning the leveling of dental arches becomes absolutely essential.
»*Inclination of maxillary incisors:* It is known that, when projecting the maxillary anterior teeth, the anterior overbite is reduced, the upper incisal line is flattened and the light reflection on the buccal surfaces of maxillary incisors is changed.^2^ ^,^ ^5^ ^,^ ^7^ ^,^ ^17^ ​​The esthetic losses in these cases are notorious and range from the perception of incisors with smaller vertical dimensions and altered proportions to a smile with a less youthful aspect (Fig 8A to E). Conversely, when the position of these teeth is planned more orthogonally in relation to the environment light beam, the perception of the actual size and proportions of maxillary incisors is allowed, as well as a more marked curvature of this incisal line (Fig 8F to J). These considerations become particularly important concerning treatments performed by previous dental expansions, either to solve lack of space, to compensate for a marked overbite or Class III malocclusion.
»*Utilization of Class II and III elastics.* It is known that the use of this mechanical resource can promote changes in the inclination of the occlusal plane. When prescribed in Class II direction, they tend to rotate the upper occlusal plane in clockwise direction, which can influence the increase in overbite and increase the perception of convexity of the upper incisal line. Conversely, elastics in Class III direction can promote counterclockwise rotation of the lower occlusal plane, tending to increase the overbite by the extrusion of mandibular anterior teeth (Figs 9A and B). This side effect requires the accomplishment of compensatory maxillary anterior teeth intrusion that are very unfavorable to the smile esthetics and speech (by reducing the exposure and increasing the buccal inclination of maxillary incisors, besides flattening the upper incisal line).

## 2 - MORPHOFUNCTIONAL DETERMINANTS

Aspects related to the anatomic and functional pattern of individuals, originated from their dentoskeletal, muscular and soft tissue characteristics.

**Figure 10:**
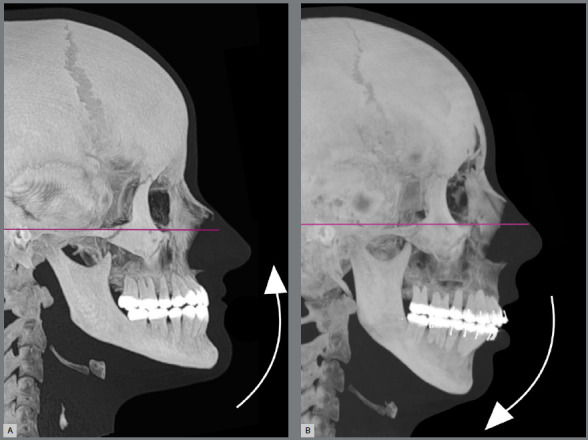
Relationship between facial archetypes and the smile arc. A) Short faces, in which there is greater tendency to counterclockwise rotation of the maxillomandibular complex, usually present occlusal plane with upward inclination, little exposure of maxillary anterior teeth when smiling and in lip rest position, more buccally inclined maxillary incisors, and a flatter or even inverted incisal line. B) Long faces, in which there is a greater tendency to clockwise rotation of the maxillomandibular complex, have opposite characteristics to those previously described and greater possibilities to achieve more pleasant smile arcs.

**Figure 11:**
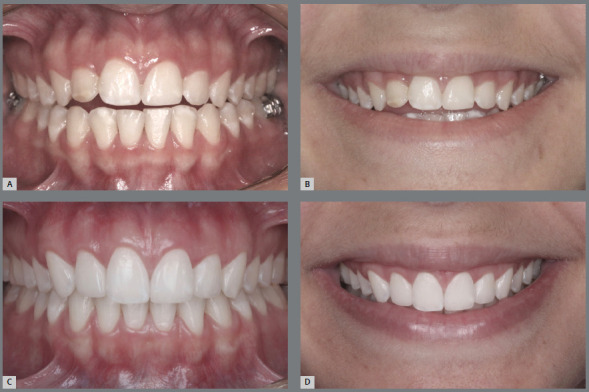
A, B) Young patient, with anterior open bite, concave upper incisal line and inverted smile arc. C, D) Upper leveling performed by the combination of posterior intrusive and anterior extrusive movements, achieving a better upper anterior incisal design. Retreatment of this case provided a pleasant smile arc, compatible with the patient’s age and personal preference.

*» Inclination of upper occlusal plane in the sagittal plane*: it is known that individuals with brachycephalic facial archetypes present occlusal and mandibular planes more inclined in counterclockwise direction in relation to the Frankfort horizontal plane (FHP), and more parallel to each other, due to a predominantly horizontal facial growth vector. Clinically, it is observed that these patients tend to have flatter upper incisal lines (or even reverse, if the maxillary bone bases present counterclockwise rotation) (Fig 10A).^2^ Though not yet scientifically evidenced, this seems to be a relevant clinical perception. The opposite may also be expected: patients with dolicocephalic facial archetypes, presenting occlusal and mandibular planes more inclined in clockwise direction in relation to the FHP, tend to have greater possibilities for the achievement and visualization of more convex and marked upper incisal lines (Fig 10B).
»*Vertical relationship of anterior teeth:* this topic includes some malocclusions that present changes in overjet and overbite, as dentoalveolar open bite, edge-to-edge incisor relationship, and increased overbite. Open bites of dentoalveolar origin (usually caused by abnormal pressure habits) have an inverted upper incisal curvature, besides minimal or no exposure of maxillary anterior teeth in lip rest position and during speech. In mild and moderate cases, the achievement of a convex upper incisal line occurs in a relatively simple and predictable manner, by the extrusion and/or retraction of maxillary anterior teeth (Figs 11 A to D).

**Figure 12:**
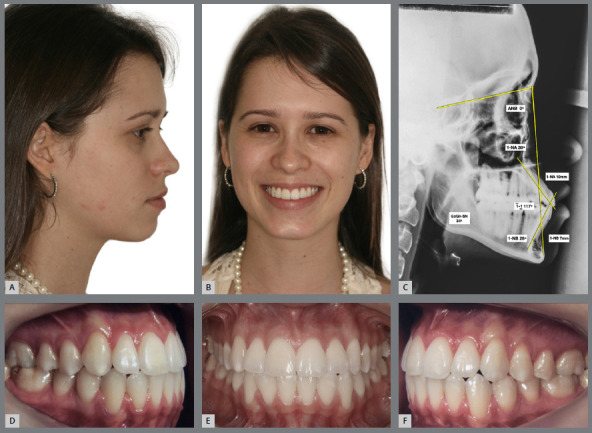
A, B) Facial photographs of patient with a slightly convex profile, lower lip protrusion and buccal inclinations of maxillary incisors. C) Initial lateral cephalogram and some Steiner cephalometric measurements, describing a skeletal Class III pattern and dentoalveolar bimaxillary protrusion. D-F) Intraoral photographs showing mild Angle Class III malocclusion, compensated by the projection of maxillary incisors, presenting acceptable posterior intercuspation, minimum values of overbite and overjet, flattening of the upper incisal line, and tooth-size discrepancy with 2-mm excess of mandibular anterior teeth width.

Conversely, individuals with minimal overbite or edge-to-edge anterior relationship require improvement of overjet and overbite. If the situation occurs due to presence of tooth-size discrepancy, with excess of lower dental tissue, the problem can be corrected by proximal stripping (obviously, if allowed by the dental anatomical and periodontal conditions), followed by retraction of mandibular incisors and extrusion of maxillary incisors (the clinical case described in Figures 12 to [Fig f13] 14 illustrates this situation). If the case involves a compensated Class III malocclusion, the formation of a convex upper incisal line and a pleasant smile arc is more difficult.

**Figure 13: f13:**
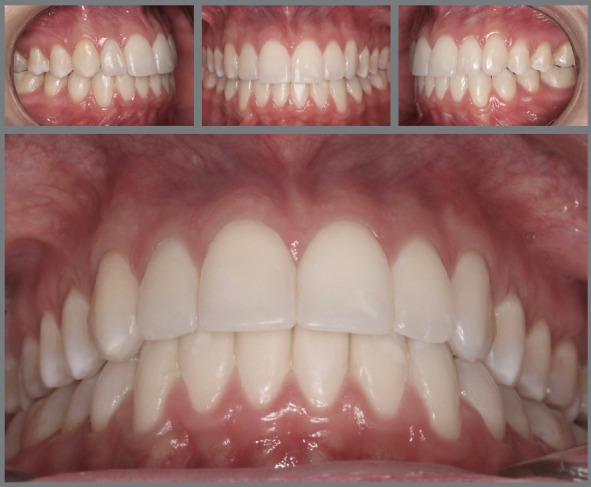
Final intraoral photographs of orthodontic retreatment, showing good overjet and overbite, and good coordination of dental arches. The desired tooth movement was allowed by proximal stripping performed in the maxillary and mandibular anterior regions, aiming at correcting the Bolton discrepancy, and improving the maxillary anterior dental proportions (increased dominance of the maxillary central incisors). Also observe the greater convexity of the upper incisal line.

**Figure 14:**
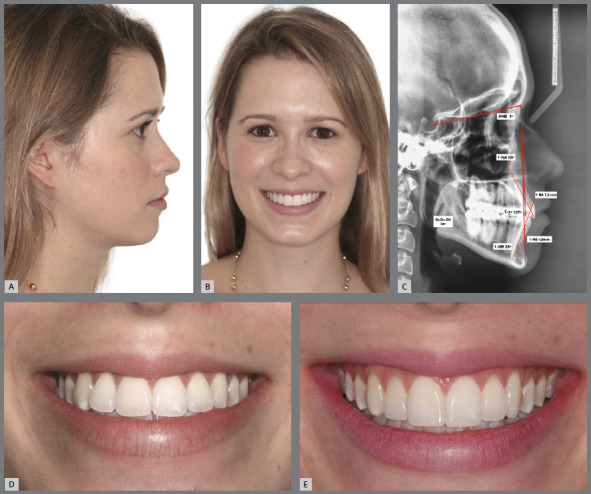
A, B) Final facial photographs, showing the reduction of lower lip protrusion and the improvement of maxillary teeth inclination. C) Final lateral cephalogram and some Steiner cephalometric measurements, showing the reduction of dentoalveolar bimaxillary protrusion. Close-up photographs of the initial (D) and final (E) smile, showing the esthetic improvement obtained after disinclination and extrusion of maxillary incisors, increase of gingival exposure and upper incisal line curvature, with consequent achievement of a more beautiful and youthful smile arc.

**Figure 15:**
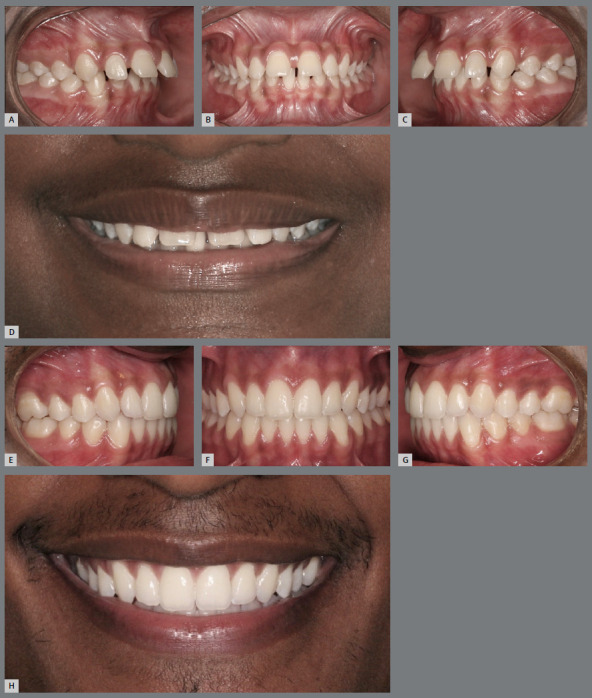
Intraoral and smile photographs, initial (**A-D**) and final (**E-H**), showing a case of marked overbite and upper dentoalveolar protrusion. The improvement in upper incisal line curvature was obtained by extrusion and displacement of maxillary incisors. For that purpose, it was necessary to perform an excellent leveling of the mandibular arch.

The increased overbite may be observed in all types of malocclusions, and its presence is associated with some overjet, either positive (Class II or due to the presence of tooth-size discrepancy) or negative (Class III). Two important aspects should be understood:

1. The strategies for correction are determined by the exposure of maxillary incisors in dynamic analysis (lip position at rest and during speech).^1^ ^,^ ^2^ ^,^ ^5^ ^,^ ^9^ ^,^ ^10^ ^,^ ^11^ ^,^ ^13^ ^,^ ^17^ ^,^ ^20^ If it is necessary to maintain or improve this important esthetic aspect, maxillary anterior teeth intrusion should not be performed. Also, for example, the use of “steps” in continuous or utility archwires is considered a very unfavorable mechanical strategy for overbite correction, when it is desired to maintain or achieve pleasant and youthful upper incisal lines.^3^ ^,^ ^12^ ^,^ ^13^ ^,^ ^14^
  Analyzing the “time” factor, the expected reduction of maxillary teeth exposure and the tendency to flattening of the upper incisal line - resulting from dentofacial aging - should always be remembered. Excessive appearance of maxillary incisors during speech and in lip rest position, as well as excessively marked incisal curvatures, in older individuals, may not be welcome. Patients become esthetically more natural when their dentofacial characteristics are as expected for each stage of their lives.^3^ ^,^ ^4^ ^,^ ^5^ ^,^ ^6^ ^,^ ^10^ ^,^ ^13^ ^,^ ^18^
2. Clinically, it seems that cases of overbite more favorable to maintaining or achieving a beautiful smile arc are those that can be corrected by mandibular anterior teeth intrusion and/or mandibular posterior teeth extrusion (Fig 15).

When marked overbite is associated with a brachycephalic skeletal pattern, in a patient with short face and horizontal occlusal and mandibular planes or even with counterclockwise rotation, there is an esthetic problem with difficult orthodontic solution. Usually, the improvement of maxillary teeth exposure and the possibility to achieve a convex and pleasant upper incisal line requires an orthodontic-surgical approach, with downward replacement of the maxilla and clockwise rotation of the aforementioned planes.

»*Incisal wear:* the wear of edges of maxillary anterior teeth can be diagnosed at different ages, even though they are more common in adult patients. They can be physiological and considered as “marks” of the functional activity of teeth, or they may indicate substantial loss of coronal tissue, most often caused by perimolysis and/or eccentric bruxism.^23^

**Figure 16:**
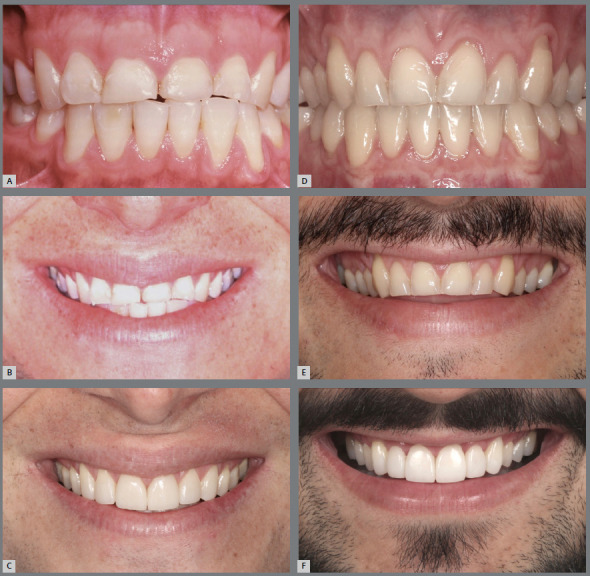
Initial (**A, B; D, E**) and final (**C, F**) intraoral and smile photographs of two young patients with anterior “edge-to-edge” relationship, incisal wear, changes in volume and dental proportions and gingival contour of the maxillary anterior region, inverted curvature of the upper incisal line and reverse smile arc. Both patients were orthodontically treated to obtain adequate levels of overjet and overbite and restoration of worn teeth.

Besides changing the size of teeth and their esthetic proportions, the wear changes the design of incisal lines and consequently the perception of the smile arc (Fig 16A to F). In some situations, they are followed by compensatory extrusion of incisors and changes in gingival contour and in the “pink esthetics” (Fig 16B). These patients should be treated, ideally and conservatively, by a transdisciplinary approach in which orthodontics works in partnership with restorative specialties.^23^ ^-^ ^27^

**Figure 17:**
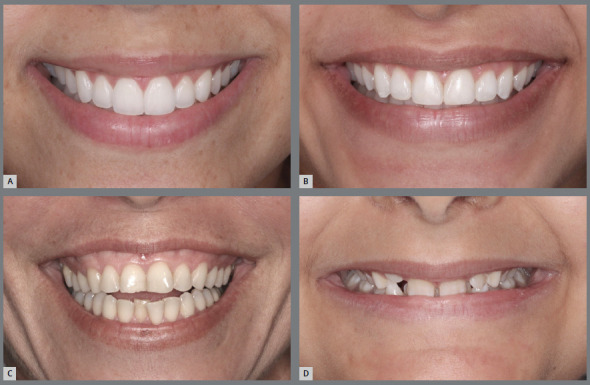
The behavior of lips, during their “unveiling”, has a favorable influence on the formation of smile arc of types “Monalisa” and “Canine” (**A,** B). In smiles of the “Complex” and “Infinite or mirror” type, it is more difficult to achieve harmony between the upper incisal line and the lower lip. In these cases, Orthodontics faces its major limitation: alteration of the muscle and tissue pattern of the lips and perioral regions.

»*Lip design, when smiling:* Since the smile arc is an esthetic parameter that depends on symmetry and consonance between two curved lines with the same orientation - the upper incisal curvature and the line formed by the lower lip when smiling -, it is possible to understand the orthodontic limitation in the process of achieving beautiful results for all patients. Even with upper incisal lines with youthful and pleasant curvatures, the behavior of lips and commissures during smile is determined by the contraction pattern of perioral muscles and their morphological characteristics (volume, thickness, symmetry). That is to say, it seems that Orthodontics has little or no resources to change these characteristics. It is possible to observe some types of smile classified and described in the literature that favor or not the appearance of the smile curve. The most favorable are the Monalisa and Canine^18^ types (Figs 17A and B), in which the commissures are moved upward, and the lower lip forms a curve accompanied by the upper incisal design. This is more rarely observed in smiles of Complex and Infinite (or mirror) types,^2^ ^,^ ^16^ for example (Figs 17C and D).

## 3 - POSTURAL AND RECORDING DETERMINANTS

These are related to possible changes in the visual perception of the smile arc, as well as its recording.^2^

**Figure 18:**
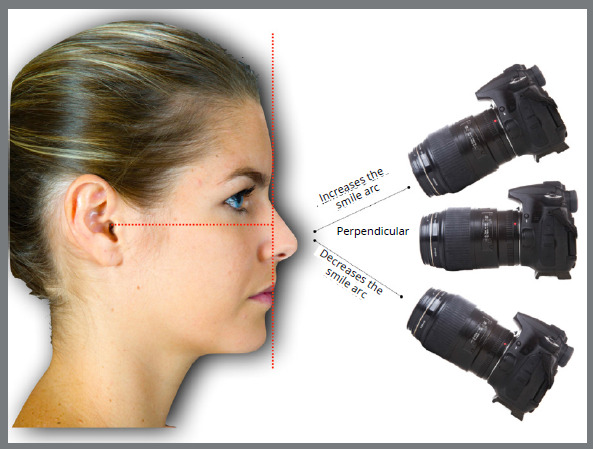
Natural head position (NHP) and imaginary frontal line parallel to it. The camera should be positioned perpendicular to the latter.

»*Change in patient’s head position:* The orthodontic photographic images should take as reference a position with good reproductive accuracy, which is the natural head position (NHP). The camera should be parallel to the latter and perpendicular to the true vertical line (imaginary line that crosses the line of view perpendicularly, dividing the face into right and left sides) (Fig 18).

**Figure 19:**
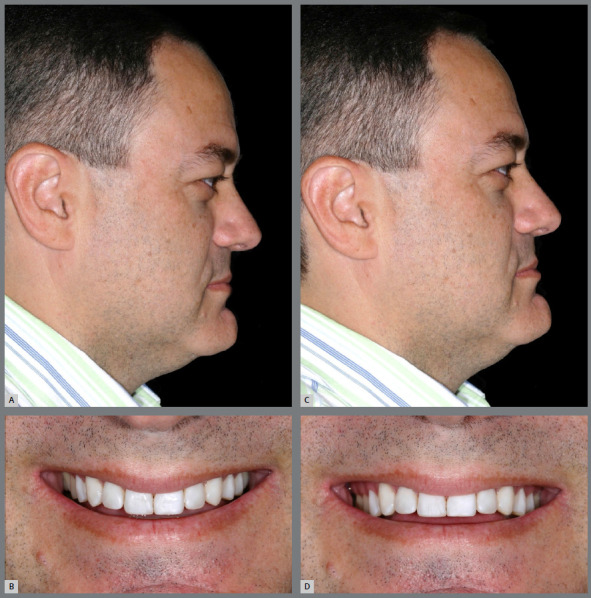
Changes in inclination of the patient’s head during photographic recording: **A,**B) By inclining it downwards, the occlusal plane is angulated inferiorly, and the curvature of the upper incisal line is increased. **C,** D) By inclining it backwards, the occlusal plane is angulated superiorly, and the curvature of the upper incisal line is flattened or even inverted.

By inclining the patient’s head slightly up or down, it is also inclined in relation to the camera and, consequently, the visual perception of the smile arc curvature is changed. The described movement modifies the angle of the dental occlusal plane in relation to the observer, leading to a perception of flatter or more curved upper incisal line (Fig 19). The downward inclination of the head, together with the parallax factor, potentiates the formation of the smile arc. This condition needs to be carefully evaluated to avoid masking structural problems. The opposite is also true; the perception of the esthetic effect of correct dental leveling can be impaired by inclining the head upwards.

**Figure 20:**
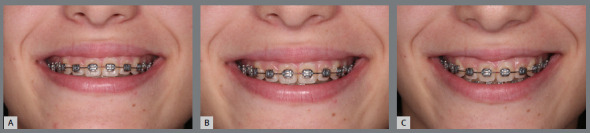
Changes in perception of the smile arc, achieved with different camera inclinations in relation to the occlusal plane. A) Camera inclined upwards, planning the upper incisal line curvature. B) Camera parallel to the occlusal plane, allowing correct analysis of the smile arc; and C) Camera inclined downwards, increasing the upper incisal line curvature.

»*Inclination of photographic camera:* alike the change in position of the patient’s head can modify the visualization of the smile arc, the position of the camera that records the smiles may also influence. Photographs taken with cameras angled from top to bottom produce images with more marked incisal and lip curvatures, while cameras angled from bottom to top may flatten them (Fig 20).

To record the smiles as accurately and reproducibly as possible, it is necessary to pay attention to this aspect and attempt to achieve the photographs in a manner as careful and standardized as possible.

## FINAL CONSIDERATIONS

The achievement of beautiful, natural and pleasant smiles has a direct correlation with the dental, skeletal and facial characteristics of each individual. Also, the artistic perception of the orthodontist, the individualization of the appliance placement/digital setup, as well as the knowledge on orthodontic mechanics, can favor or impair the treatment outcomes. The smile arc is one of the most important esthetic parameters for dentistry and should receive special attention in contemporary orthodontic planning. Understanding the factors that determine its appearance is essential for maximum use of its esthetic potential.
